# VA/VHAG alternating consolidation regimen combined with umbilical cord blood and mesenchymal stem cell infusion for the treatment of high-risk MDS patients who are unfit for transplantation: a case report and literature review

**DOI:** 10.3389/fonc.2026.1708480

**Published:** 2026-04-22

**Authors:** Qixin Sun, Zhenzhen Wen, Yanying Ling, Jiongxiang Kuang, Guiping Chen, Zhigang Zhu, Hong Wang, Kangbao Li

**Affiliations:** 1 Department of Geriatric Hematology and Oncology, Guangzhou First People’s Hospital, Guangzhou, Guangdong, China; 2 Blood Purification Center, Guangzhou First People’s Hospital, Guangzhou, Guangdong, China; 3 Clinical Laboratory Department, Guangzhou First People’s Hospital, Guangzhou, Guangdong, China; 4 Department of Trauma and Orthopedics, Guangzhou First People’s Hospital, Guangzhou, Guangdong, China; 5 Department of Geriatric Gastroenterology, Guangzhou First People’s Hospital, Guangzhou, Guangdong, China

**Keywords:** mesenchymal stem cell, myelodysplastic syndrome, treatment, umbilical cord blood, venetoclax

## Abstract

The only treatment option for high-risk MDS patients unfit for transplantation is continuous treatment with a hypomethylating agent (HMA). In this context, some new combination HMA regimens and other drugs with different mechanisms of action have been developed recently; however, no breakthroughs have been made. In our clinical practice, we have gradually explored various new methods—induction therapy with a modified VIALE-A (VA) regimen and an alternating regimen of modified VA and VHAG for the consolidation period. Some patients who were persistently positive for MRD were treated with HLA-mismatched umbilical cord blood, and patients with poor hematopoietic recovery additionally received mesenchymal stem cells infusion. Together, these treatments showed good efficacy. Here, the total treatment process for a typical patient is described, and a review of the literature is provided.

## Background

**Figure 1:**
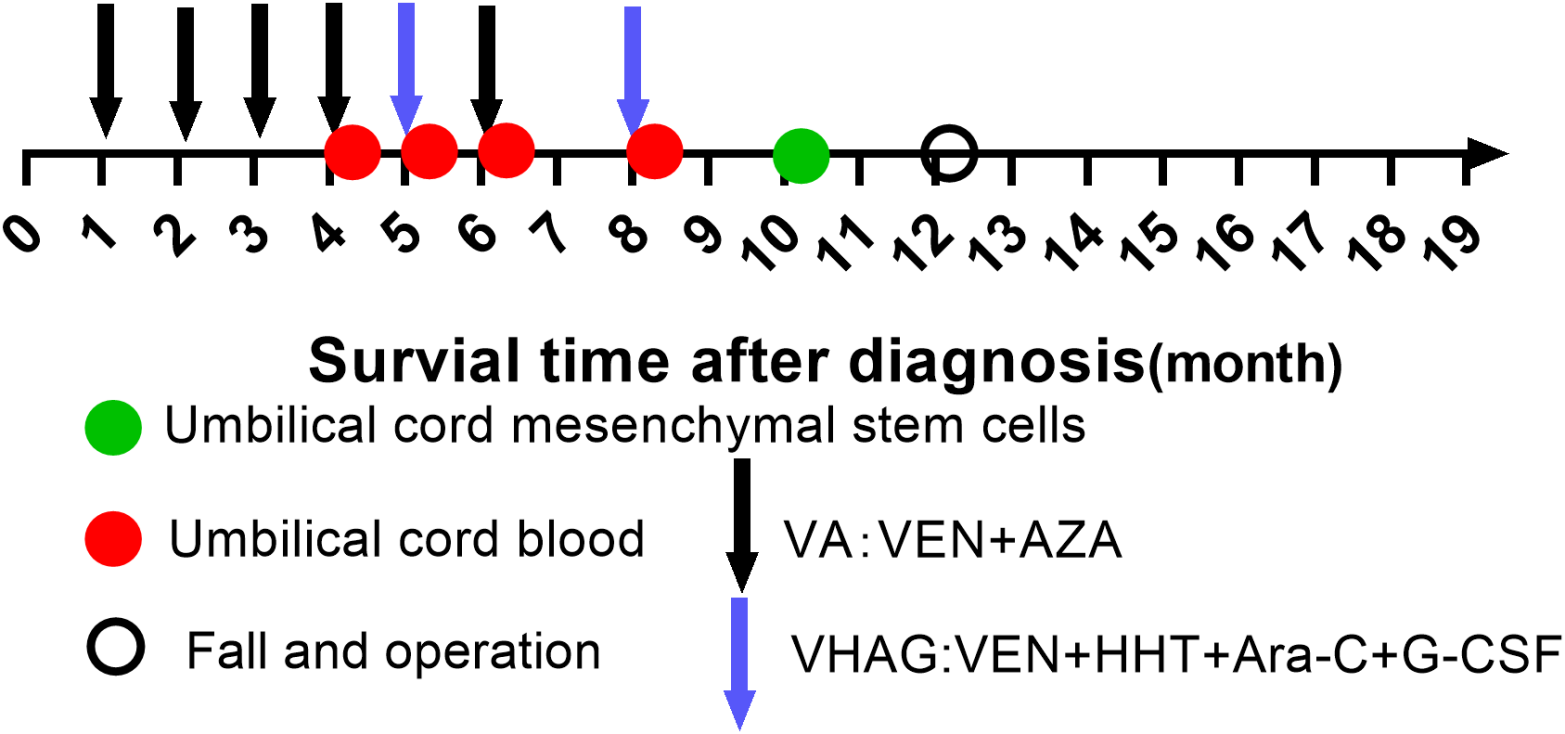
Course of treatment. After diagnosis, black arrows indicate administration of venetoclax (VEN) plus azacitidine (AZA); blue arrows indicate the VHAG regimen (VEN, homoharringtonine [HHT], cytarabine [Ara-C], and granulocyte colony-stimulating factor [G-CSF]); green circles indicate infusion of umbilical cord mesenchymal stem cells; red circles indicate infusion of umbilical cord blood; and an open black circle indicates a fall and surgery.

The first-line treatment for high-risk myelodysplastic syndrome (MDS) patients is hematopoietic stem cell transplantation or continuous treatment with a hypomethylating agent (HMA) until the disease progresses or no further benefits are obtained (1, 2). The intensity and frequency of HMA treatment can be adjusted according to the patient’s blood counts and tolerance (3, 4). In our center, based on continuous azacitidine treatment, we combined azacitidine and low-dose venetoclax to create a modified VIALE-A (VA) induction phase regimen for high-risk MDS patients who were unfit for transplantation and alternately administered this regimen and the VHAG regimen to patients in the consolidation stage. Some patients (persistently MRD-positive patients) were also given HLA-mismatched umbilical cord blood cells and mesenchymal stem cells (MSCs) infusions. Together, these regimens achieved good therapeutic effects. Here, the complete treatment process for a typical patient is summarized (see **Figure 1**).

## Case presentation

A 77-year-old woman presented to our clinic in May 2023 with progressive weakness over the previous six months. Routine peripheral blood tests revealed hemocytopenia, and the WBC count, Hb level and PLT count were 1.46×10^9^/L, 42 g/L and 20×10^9^/L, respectively. Considering the possibility of a hematological disease, she was admitted to the hospital for further treatment. A series of bone marrow tests were performed according to the 2016 WHO classification of myeloid neoplasms (5).

Bone marrow aspirate smear revealed 2.5% immature cells. BM biopsy revealed hypercellularity, and immature megakaryocytes characterized by small round nuclei were easily visualized (MF-0). Chromosome analysis failed because no metaphase cells were observed. Fluorescence *in situ* hybridization (FISH) analysis revealed that 3.4% of cells had a gain of chromosome 8 and 15% of cells had a deletion of chromosome 5q. Immunophenotyping revealed that 3.8% of immature myeloid cells had an immunophenotypic abnormality. NGS revealed TP53 gene mutations, with a VAF of 18.9% and 19.2% in exon 4 and exon 5, respectively. On the basis of these results, the patient was diagnosed with MDS-MLD. The IPSS-R score was 5, and the IPSS-M score was 2.42 (very high).

The patient was treated in our center with a modified VA regimen (Azacitidine, AZA 75 mg/m^2^, from d1-7; venetoclax, Ven 200 mg/d from d8-28) two weeks after diagnosis. The planned frequency was approximately once a month. Complications such as lung infection, pleural effusion and anemia were routinely treated during the chemotherapy period. The whole therapeutic process went smoothly. Severe organ dysfunction and fungal infection were not observed. Three cycles later, the patient’s hemocytopenia completely recovered, but flow cytometry (FCM) analysis of a BM sample still revealed minimal residual disease (MRD) positivity. To decrease MRD and delay the development of resistance to the modified VA regimen, we chose to use the VHAG regimen (Homoharringtonine, HHT 0.6 mg/m^2^/d from d1-7; Cytarabine, Ara-C 25 mg/m^2^/d from d1-7; G-CSF 5 mg/kg/d from d1-7; Venetoclax, Ven 200 mg/d from d8-28) and modified VA regimen alternately in the subsequent maintenance period. One unit of HLA-mismatched (1/10 HLA match) umbilical cord blood (UCB) of the same blood type was infused after each subsequent chemotherapy cycle. IL-2 was subcutaneously injected after infusion for 7 consecutive days. A total of four umbilical cord blood units were given to this patient. MRD was successfully reduced to <0.01%. However, a new problem was encountered—bone marrow hematopoietic recovery was delayed and the patient became transfusion dependent, especially with respect to platelets. After waiting for several weeks, to promote the rapid recovery of bone marrow hematopoiesis, we decided to give the patient a unit (5*10^7^ cells) of umbilical cord mesenchymal stem cells (MSCs). Only one week later, the platelet counts of this patient stabilized at 30–50*10^9^/L, and she was discharged and underwent regular follow-up. Unfortunately, approximately one month later, this patient fell and fractured her right femoral head, which required bone surgery. To date, the MDS therapy plan has been interrupted for twenty months. The patient can walk with the aid of a walker, and blood test results show that she is still transfusion independent, with hematopoietic growth factors administered only occasionally.

## Discussion

The outcomes for high-risk MDS patients who are unfit for transplantation are still unsatisfactory. HMAs (azacitidine or decitabine) remain the first-line recommended treatment for these patients (6). Although the median survival time has been reported to be 24.5 months in multicenter clinical trials (7), real-world studies have revealed that the median survival time is approximately 15~18 months (8–10).

To delay resistance and further increase overall survival, MRD should be reduced as low as possible. Several studies on HMA combined with venetoclax (11, 12) or anti-TIM3 or anti-47 monoclonal antibody have been carried out (13, 14), and the results have been promising, but none of these drugs have been approved for high-risk MDS patients who are unfit for transplantation (15–17). In our center, therapeutic exploration for these patients is based on continuous treatment with azacitidine combined with low-dose venetoclax to form a modified VA regimen. We found that this adjustment better balances efficacy and adverse events; however, once disease progression occurs, we face the dilemma that no effective salvage plan is available.

HAG (including Homoharringtonine, Cytarabine, G-CSF) was a widely used regimen for high-risk MDS patients in China before the HMA era, and an increasing number of studies have shown the synergistic effect of HAG combined with HMA or venetoclax (18, 19). We combined HAG with venetoclax to form a VHAG regimen to treat patients resistant to the modified VA regimen. To date, more than 10 patients have been treated, and all achieved CR2 (data not shown). Although the remission duration was limited, the advantage was that adverse events were relatively mild. We subsequently used this method and alternated it with the modified VA regimen in the consolidation phase. The advantage of this alternating consolidation regimen is that it is not only based on continuous treatment with azacitidine, but also increases the cross-use of chemotherapy drugs with different mechanisms of action. We speculated that, in theory, this alternating regimen could further delay drug resistance.

Venetoclax is the most commonly used drug in combination for the treatment of high-risk MDS, but two problems are encountered in its off-label usage. The first problem is that the suitable dosage for MDS is not clear (20–22). None of the reported regimens are fully satisfactory in terms of therapeutic efficacy or toxic reactions. The second problem is the inevitable chemoresistance that develops after long-term maintenance therapy and the lack of an effective salvage regimen after disease progression (23, 24).

The difference between our modified VA regimen and the completed MDS VERONA study and standard VIALE-A regimen is that the dosing time is increased from 14 to 21 days (25), and the total drug dose is decreased from 300–400 mg ×14 d to 200 mg ×21 d (26). If some patients experienced severe delayed bone marrow recovery, the dosage was permitted to be further reduced to 200 mg ×14 d. We found that all the patients tolerated this adjustment well. The early data from our small-sample retrospective analysis revealed better efficacy than that of HMA monotherapy, but compared with the results of the VERONA study (25), the true efficacy remains unclear.

In addition, for some patients who were persistently FCM- MRD positive or showed a gradual FCM-MRD increase (as was the case in this patient), we combined HLA-mismatched umbilical cord blood cell infusion with IL-2 injection to further reduce MRD through a weak graft-versus-leukemia (GVL) effect.

In fact, during the cumulative total of more than 10 infusion processes performed at our center, we found that no one developed grade I-IV GVHD, with the exception of fever, and the FCM-MRD of most patients could be reduced to varying degrees through several umbilical cord blood infusions (date not shown). However, we also found that some patients experienced a decrease in bone marrow proliferation or delayed hematopoietic recovery, which led to transfusion dependence, sometimes similar to that seen in patients with aplastic anemia. Therefore, we administered umbilical cord MSCs to these patients to promote hematopoietic recovery. In this case, the efficacy of this treatment was particularly obvious; this patient was discharged from the hospital only one week after transfusion. Although the patient fell and fractured her femoral head while traveling, her physical condition enabled her to tolerate general anesthesia and total hip arthroplasty well. To date, she is still alive (36 months after diagnosis) and transfusion- independent, requiring only intermittent administration of hematopoietic factors. This result far exceeds the median survival time (12 months) predicted by IPSS-M risk calculator at the time of diagnosis. In addition, reflecting on the entire treatment process also provides us with some insights. For high-risk MDS patients, if the initial treatment intensity is adequate and MRD testing is persistently negative, it appears that gradually extending the treatment interval and/or transitioning to best supportive care within a certain period of time is appropriate.

The purpose of all these therapeutic regimen explorations was to reduce MRD and prolong survival as long as possible. In fact, all these measures are based on the principles and methods of transplantation for high-risk AML patients. For example, the GVL effect induced by HLA-mismatched cord blood was borrowed from donor lymphocyte infusion (DLI), which has been confirmed to reduce MRD and prevent relapse (27, 28). Umbilical cord MSCs improve the bone marrow microenvironment and regulate immunity, thereby enhancing bone marrow stromal hematopoiesis and ameliorating hematopoietic failure after transplantation (29, 30). To the best of our knowledge, there are no similar reports on the comprehensive treatment of high-risk MDS patients who were previously unfit for transplantation. Certainly, there are still some questions that are difficult to answer regarding our treatment, such as how to verify the existence of GVHD and GVL effect and how to evaluate and predict their intensity after HLA-mismatched umbilical cord blood transfusion. We found that the damage to target organs, such as the skin or intestine, and changes in the CD4/CD8 T-cell ratio that typically occur in GVHD were not significant after infusion of HLA-mismatched cord blood.

Currently, the patients we choose for infusion are mainly FCM-MRD positive or have a progressive increase in MRD. We use FCM-MRD reduction as an interim evaluation criterion to judge the efficacy after infusion. However, there are differing opinions among the International Working Group (IWG) on whether this evaluation method is appropriate for MDS patients (31).

MSCs have already been widely used in the treatment of many types of hematological diseases (30), especially for transplantation. Although most studies are retrospective, all of them have shown that transplantation combined with MSCs can promote engraftment, accelerate hematopoietic recovery, reduce the incidence of GVHD and transplantation-related complications (29, 32). Compared with bone marrow-derived MSCs, umbilical cord-derived MSCs are easier to obtain without harm to the donor, proliferate faster and have a lower level of HLA class I molecules (33, 34). Although some studies found that MSCs obtained from different parts of the umbilical cord could have different biological functions (35), the considerable advantages of the lack of ethical burden and the ease of commercial preparation make umbilical cord MSCs increasingly popular in the clinic. To date, the addition of umbilical cord MSCs to the treatment regimens of MDS patients who are unfit for transplantation has not been reported.

Finally, the efficacy of treatments for high-risk MDS patients who are unfit for transplantation is still not satisfactory. The VA/VHAG alternating consolidation regimen combined with umbilical cord blood and/or MSC transfusion, which was initiated by our center, has shown good effects; however, it should be noted that this comprehensive regimen is still a non-radical approach. Considering the small number of patients, the therapeutic effect needs to be confirmed and improved in future studies with larger sample sizes.

## Data Availability

The original contributions presented in the study are included in the article/supplementary material. Further inquiries can be directed to the corresponding author/s.
